# A bioanalytically validated RP-HPLC method for simultaneous quantification of rivaroxaban, paracetamol, and ceftriaxone in human plasma: a combination used for COVID-19 management

**DOI:** 10.1038/s41598-024-75729-y

**Published:** 2024-10-28

**Authors:** Reham A. Ismail, Miriam F. Ayad, Lobna A. Hussein, Yossra A. Trabik

**Affiliations:** Department of Pharmaceutical Analytical Chemistry, Faculty of Pharmacy, Ain Shams University, Abbassia, 11566 Cairo Egypt

**Keywords:** Analytical chemistry, Green chemistry

## Abstract

Rivaroxaban is a direct oral anticoagulant medication that has been found to be beneficial for the management of thromboembolic events linked to the Corona virus disease 2019 (COVID-19) pandemic, which has resulted in more than 6 million deaths worldwide. Hence, a sensitive, selective, and green bioanalytically validated method was developed using RP-HPLC coupled with DAD for the simultaneous determination of rivaroxaban in human plasma, and two co-administered drugs, namely, paracetamol, an analgesic, and ceftriaxone, an antibiotic, that are used in the management of COVID-19. An Exsil 100 ODS C18 column (250 × 4.6 mm, 5 μm) was used as the stationary phase, and acetonitrile: water: methanol at a ratio of 60:30:10 (v/v/v) was used in isocratic mode as the mobile phase with a flow rate of 0.7 ml/min. The method was validated over a concentration range of 0.1–10.0 µg/mL for rivaroxaban, and 1.0–15.0 µg/mL for paracetamol and ceftriaxone. The lower limits of detection (LLODs) were found to be 0.03, 0.32, and 0.32 µg/ml for rivaroxaban, paracetamol, and ceftriaxone, respectively. Moreover, the lower limits of quantitation (LLOQs) were found to be 0.1, 0.96, and 0.98 µg/ml. The developed method showed excellent accuracy and precision for the determination of the aforementioned drugs. Four metrics were used to evaluate the greenness of the developed method. The results revealed that the suggested method is green, with values of 81 and 0.6 for the analytical eco-scale and analytical greenness assessment (AGREE), respectively.

**Supplementary Information:**

The online version contains supplementary material available at 10.1038/s41598-024-75729-y.

## Introduction

Coronavirus disease 2019 (COVID-19) is a highly contagious viral sickness brought on by the SARS-CoV-2 virus, which causes severe acute respiratory syndrome. It had a devastating impact worldwide, causing more than 6 million deaths globally^1^. According to the report of the first 425 confirmed cases in Wuhan, the symptoms that are most frequently reported include fever, myalgia, dry cough, and exhaustion^1^. In addition to the previous symptoms, a higher incidence of thromboembolic illness has also been reported in SARS-CoV-2 patients. It can manifest as cutaneous thrombosis, pulmonary embolism, stroke, or coronary thrombosis^2^. Several antithrombotic medications, such as direct oral anticoagulants (DOACs), have been suggested as possible prophylaxis and treatments to avoid COVID-19-associated thrombosis^3^. Rivaroxaban, 5-chloro-N-[[(5 S)-2-oxo-3-[4-(3-oxomorpholin-4-yl) phenyl]-1,3-oxazolidin-5-yl] methyl] thiophene-2-carboxamide, is a DOAC that can be used for the prevention and treatment of thromboembolic events. It is a highly specific and reversible inhibitor of factor Xa (FXa) that prevents the interaction of prothrombin with coagulation cascade components and reduces the risk of thromboembolic events^3^. In addition to anticoagulants, other therapies are needed to control other symptoms, as supportive treatments such as analgesics and antibiotics^4^. Supportive treatments, such as paracetamol, N-(4-hydroxyphenyl) acetamide, a non-steroidal anti-inflammatory drug (NSAIDs) has been suggested for controlling other symptoms such as pain and high temperature, in patients with COVID-19^5^. Additionally, utilizing antibiotics, such as ceftriaxone, (6R,7R)-7-[[(2Z)-2-(2-amino-1,3-thiazol-4-yl)-2-methoxyiminoacetyl]amino]-3-[(2-methyl-5,6-dioxo-1 H-1,2,4-triazin-3-yl)sulfanylmethyl]-8-oxo-5-thia-1-azabicyclo[4.2.0]oct-2-ene-2-carboxylic acid, is advised for COVID-19 patients experiencing sepsis, as well as for moderately, severely, and critically ill COVID-19 patients^6^. Several chromatographic methods have been developed for determination of rivaroxaban in its dosage form and plasma^7–12^ as well as for determination of paracetamol^13–18^ and ceftriaxone in pharmaceutical formulation and biological fluids^19–23^. However, to the best of our knowledge, no chromatographic method has been published for simultaneous determination of rivaroxaban together with paracetamol and ceftriaxone as co-administered drugs for Covid-19 management in human plasma. Therefore, the goal of this work was to develop an easy-to-use, fast, and environmentally friendly method for detecting and measuring the levels of our analytes (rivaroxaban, paracetamol, and ceftriaxone) in human plasma using HPLC technique. The developed HPLC method was bioanalytically validated in accordance with FDA guidelines. The greenness of the suggested method was evaluated using four green metric tools: national environmental methods index (NEMI), analytical eco-Scale (ESA), green analytical procedure index (GAPI), and analytical greenness index (AGREE).

## Experimental

### Material and reagents

#### Pure standards

**Fig. 1:**
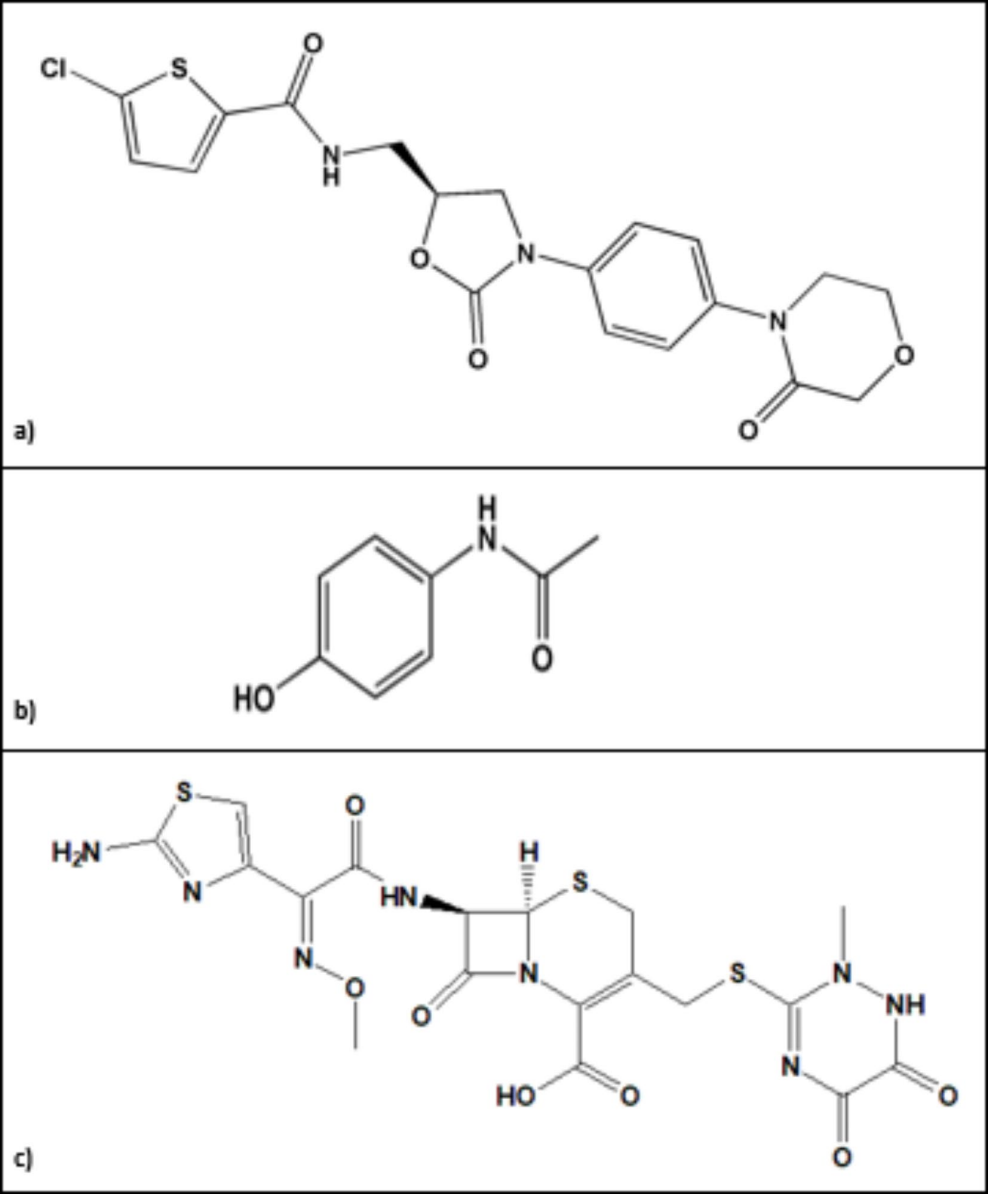
Chemical structures of the studied compounds: (**a**) rivaroxaban, (**b**) paracetamol, and (**c**) ceftriaxone (drawn by chem bio draw ultra).

El-Nasr Pharmaceutical Chemicals Company (Qalyubia, Egypt) generously donated paracetamol. Eva Pharma (Cairo, Egypt) graciously provided rivaroxaban and ceftriaxone. The purity of each standard was confirmed to be at least 98.5%. Figure 1 shows the chemical composition of the tested analytes.

#### Reagents

Acetonitrile and methanol were purchased from Sigma‒Aldrich (Cornell Lab, Cairo, Egypt). Water was purchased from Fisher Scientific (Cornell Lab, Cairo, Egypt). All the solvents used were HPLC grade. Human plasma samples were obtained from VACSERA (Giza, Egypt) and stored at − 4 °C.

### Methods

#### Instrumentation and HPLC conditions

A VWR^®^ Hitachi Chromaster HPLC-DAD system with an autoinjector, quaternary pump, vacuum degasser, and Hitachi Chromaster 5430 diode array detector was used to carry out the chromatographic analysis of the pharmaceuticals under investigation. A Dr Maisch GmbH Exsil 100 ODS C18 column (250 × 4.6 mm, 5 μm) from Ammerbuch, Germany, was employed as the analytical column. At room temperature and an isocratic flow rate of 0.7 mL/min, analysis was carried out using acetonitrile: water: methanol (60:30:10; v/v/v) as the mobile phase with an injection volume of 20.0 µL. All the solvents were filtered through a 0.22 μm membrane filter. For the quantitative determination of paracetamol and rivaroxaban, the analytical wavelength was 250.0 nm, whereas it was 280.0 nm for ceftriaxone.

### Procedures

#### Preparation of standard and working solutions

Paracetamol, rivaroxaban, and ceftriaxone stock standard solutions were made in methanol at a concentration of 100.0 µg/ml each, by precisely weighing 0.01 g of each analyte; the solutions were subsequently placed separately into 100-mL volumetric flasks before the volume was completed with methanol. To create calibrator and quality control (QC) samples, standard working solution mixtures were created by mixing the proper volumes of the three stock solutions (100.00 µg/mL) into a 10-mL volumetric flask. The produced standard solutions were refrigerated at a temperature of 4 °C.

#### Preparation of calibrator and quality control (QC) samples

A 10-mL volumetric flask containing blank human plasma was spiked with the correct volumes of the three stock solutions to obtain final concentrations of 1, 2, 5, 10, 11 and 15 µg/mL for paracetamol; 0.1, 0.5, 1.0, 1.5, 3.0, 8.0 and 10.0 µg/mL for rivaroxaban; and 1, 2, 5, 7, 10 and 15 µg/mL for ceftriaxone, these concentrations were used as calibrator samples. To obtain QC samples, lower limit of quantification (LLOQ), low QC (LQC), mid QC (MQC), and high (HQC) samples were prepared at concentrations of 1.0 ,3.0 ,7.5 and 12.5 µg/mL for paracetamol and ceftriaxone, and at concentrations of 0.1, 0.3, 5.0 and 7.5 µg/mL for rivaroxaban. These solutions were maintained in a refrigerator at 4 °C when not in use.

#### Sample preparation

For sample preparation, the protein precipitation technique was used. A total of 1.0 mL of plasma (blank or spiked) was vortexed with 4.0 mL of methanol in Eppendorf tubes. The tubes were then centrifuged at a speed of 6000 rpm for twenty minutes. Afterwards, the supernatant was dried by evaporation and reconstituted in 1.0 mL of the mobile phase.

### Method validation

According to the US FDA criteria, bioanalytical validation was applied to the following parameters: linearity, accuracy, intraday and interday precision, selectivity, lower limit of quantification (LLOQ), carryover, matrix effect and stability^24^.

#### Linearity and calibration curve

For the calibration curve, at least six different standard concentrations were produced in blank human plasma, and each concentration was evaluated five times. The sample was injected following sample preparation and under the previously mentioned HPLC conditions. The average peak area of five measurements at a given concentration was then plotted against that concentration, and the regression equations were subsequently computed. Except for the LLOQ, where the acceptance threshold was 20%, each concentration level had a tolerance of 15% deviation from the nominal value. The aforementioned conditions must be satisfied by 75% for each validation run.

#### Limit of detection (LOD) and limit of quantification (LOQ)

LOD and LOQ were calculated using the following formulas:

LOD = 3.3 x σ/S.

LOQ = 10 x σ/S.

where S is the slope of the calibration curves for the medications under study, and σ is the standard deviation of the intercept.

#### Accuracy and precision

For intraday and interday accuracy and precision, five replicates at each of the four QC levels (LLOQ, LQC, MQC, and HQC) were examined on the same day and on three further consecutive days. Accuracy was evaluated using percentage recoveries. The acceptance threshold for each concentration level was a 15% departure from the nominal value, except for the LLOQ, which had a threshold of 20%. The relative standard deviation (%RSD) used to evaluate intraday and interday precision was 15% for the quality control (QC) levels, but 20% for the LLOQ.

#### Selectivity

The ability of the analytical procedure to distinguish between the analytes of interest and any matrix-interfering elements is emphasized by selectivity. The results were evaluated by comparing the chromatogram of unspiked plasma with the chromatogram of the plasma matrix spiked at the LLOQ level. The chromatogram of the spiked plasma was compared to that of blank plasma taken from six different sources. The peak areas of coeluting substances should not be more than 20% of the analyte at the LLOQ.

#### Carryover and matrix effects

Carryover was used to determine whether a sample with the highest concentration would affect the analysis of a later sample. A concentration within the upper limit of quantification (ULOQ) was injected before injecting blank plasma. After that, the peak regions of the analytes were calculated, and they should be less than 20% of the LLOQ. Matrix effects were examined to determine how endogenous substances will impact the analysis. The LQC and HQC samples were made of plasma from six separate human batches, and the peak area for the analyte of six different LQC and HQC samples was calculated to evaluate whether the %RSD was within 15% or not.

#### Stability

Stability in plasma was assessed using three − 20 °C freeze-thaw cycles. The plasma samples were kept at room temperature for 8 h to evaluate bench-top stability. The bench-top and freeze‒thaw stabilities of the three plasma-based analytes were assessed at the LQC and HQC levels. The average recovery percentages of three measurements of each analyte were subsequently determined. For the stability to meet the acceptance criteria, the calculated concentration percentage change must be less than 15% of the theoretical concentration.

### Method application in spiked human plasma

To 1.0 ml of human plasma in a set of Eppendorf tubes, various amounts of each analyte were added from their stock solutions, which were subsequently vortexed with 4.0 mL of methanol. The tubes were centrifuged for 20 min at a speed of 6000 rpm. The supernatant was then dried by evaporation and reconstituted in 1.0 mL of the mobile phase to generate the following concentrations of paracetamol, rivaroxaban, and ceftriaxone: (4.0, 8.0, 13.0) µg/mL, (0.2, 4.0, 7.0, 9.0) µg/mL, and (4.0, 8.0, 13.0) µg/mL, respectively.

## Results and discussion

Several analytical methods for the detection and quantification of rivaroxaban^7–12^, as well as for determination of paracetamol^13–18,25^, and ceftriaxone in pharmaceutical formulation and biological fluids^19–23^ have been developed in recent years. However, the current study proposes, for the first time, a sensitive and specific HPLC-UV method for the quantitative detection of rivaroxaban in the presence of additional co-administered drugs, paracetamol, an analgesic, and ceftriaxone, an antibiotic, which aid in the management of COVID-19.

### Analytes separation

A variety of mobile phases, including binary and ternary mixtures containing varying ratios of methanol, water, isopropanol, and acetonitrile, were investigated. Findings revealed that the optimal conditions for the developed technique was isocratic elution with acetonitrile: water: methanol in ratio of (60:30:10; v/v/v), which produced a symmetric peak shape and an adequate baseline separation. Acetonitrile, and methanol are the most popular organic solvents used with water in RP-HPLC applications. They have several advantages including complete miscibility with water, relatively low viscosity of their aqueous solutions, low UV cut-off wavelength (190 nm and 205 nm for acetonitrile and methanol, respectively), and low chemical reactivity with most sample species, with HPLC instrument and column surfaces^26^. The amount of water in the mobile phase was decreased to 30% to improve the retention of compounds that were weakly retained as PARA. Analysis was carried out at the selected wavelength 250.0 nm for rivaroxaban and paracetamol since they had maximum absorption at this wavelength. Measuring at this selected wavelength enhances the sensitivity of the proposed method, which agrees with previously published papers^27,28^. Wavelength 280.0 nm was chosen for ceftriaxone which complies with previously published articles^21,29,30^, and it was found that ceftriaxone has maximum absorption at this wavelength, during the practical work.

**Fig. 2:**
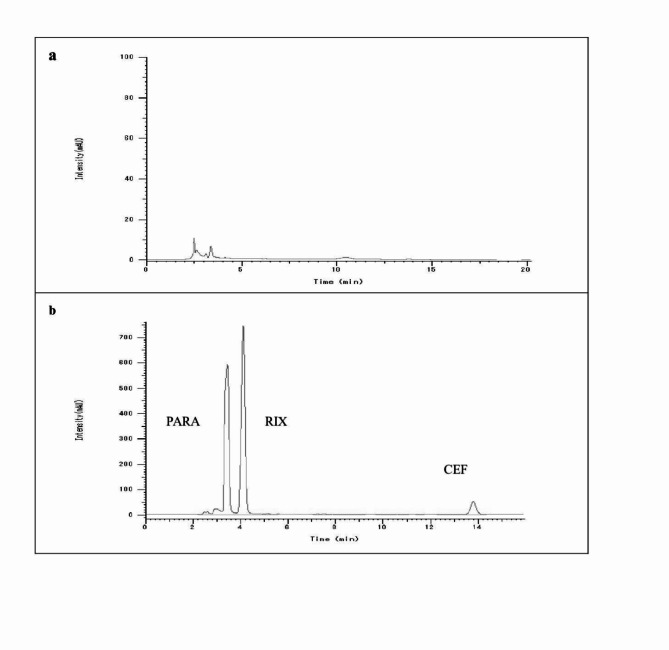
HPLC chromatogram of (**a**) blank plasma, (**b**) plasma spiked with 10 µg/mL of paracetamol, 3 µg/mL of rivaroxaban and 7 µg/mL of ceftriaxone.

Different flow rates were examined. Flow rate of 0.7 mL/min was optimum for better separation between rivaroxaban and paracetamol and to achieve short analysis time. Retention times were found to be 4.1, 3.5, and 13.8 min for rivaroxaban, paracetamol, and ceftriaxone, respectively. The created approach successfully isolated and quantified the three investigated analytes in plasma, as depicted in Fig. 2a and b.

### Bioanalytical validation of the HPLC method

According to FDA regulations, the procedure underwent bioanalytical validation, which considered the validation criteria of linearity, accuracy, precision, selectivity, carryover, matrix effect, and stability.

#### Linearity and range

The examined analytes displayed linear calibration curves with regression coefficients (r) ≥ 0.999 for concentrations of 1.0–15.0 µg/ml for paracetamol and ceftriaxone and 0.1–10.0 µg/ml for rivaroxaban. A calibration curve was drawn for each analyte, while the regression coefficient (r), slope, intercept and other validation parameters were computed, as shown in Table 1.

#### Limit of detection (LOD) and limit of quantification (LOQ)

The results, as shown in Table 1, demonstrated that the suggested method had an appropriate LOQ and LOD, together with a sufficient sensitivity. Values for LOD were found to be 0.03, 0.32, and 0.32 for rivaroxaban, paracetamol, and ceftriaxone, respectively. Values for LOQ were found to be 0.10, 0.96, and 0.98 for rivaroxaban, paracetamol, and ceftriaxone, respectively.

**Table 1:** Validation parameters of the investigated analytes in human plasma.

Parameter	Analyte		
	Paracetamol	Rivaroxaban	Ceftriaxone
Retention time (min)	3.5	4.1	13.8
Linear range (µg/mL)	(1.0–15.0)	(0.1–10)	(1.0–15.0)
Slope	127,983	1,000,000	10046.32
Intercept	93,954	741,835	− 3134.68
LOD (µg/mL)	0.32	0.03	0.32
LOQ (µg/mL)	0.96	0.10	0.98
Correlation coefficient (r)	0.9994	0.9994	0.9990

#### Accuracy and precision

Table 2 displays the results of the developed technique for the analysis of the target analytes in human plasma in terms of accuracy (as % recovery) and precision (as %RSD). The assay values were found to be accurate and precise within the permissible variability limits for both intra- and interday accuracy and precision. The intra- and interday precisions of the analytes ranged from 0.15 to 14.58% and from 0.30 to 12.43%, respectively. The analytes’ accuracy varied from 98.32 to 103.42% and from 90.22 to 100.92% for intraday and interday data, respectively.

**Table 2:** Results of accuracy, intraday and interday precisions of the investigated analytes in human plasma.

Analyte	Concentration added (ng/ml)	Intraday precision		Interday precision	
		Mean ± SD	RSD (%)	Mean ± SD	RSD (%)
Paracetamol	1.0	103.02 ± 2.66	2.58	94.21 ± 2.49	2.65
	3.0	101.57 ± 1.22	1.20	98.39 ± 1.55	1.58
	7.5	99.77 ± 5.67	5.686	98.04 ± 1.54	1.57
	10.0	101.17 ± 0.51	0.51	95.86 ± 4.73	4.93
Rivaroxaban	0.1	98.32 ± 6.38	6.49	90.22 ± 7.06	7.83
	0.3	99.04 ± 14.44	14.58	95.08 ± 4.04	4.25
	5.0	102.35 ± 8.53	8.34	93.08 ± 5.90	6.34
	7.5	99.90 ± 0.69	0.69	92.54 ± 11.51	12.43
Ceftriaxone	1.0	102.83 ± 2.81	2.74	100.92 ± 0.31	0.30
	3.0	99.85 ± 0.15	0.15	98.36 ± 10.70	10.89
	7.5	103.42 ± 0.55	0.53	100.58 ± 4.06	4.04
	10.0	100.80 ± 1.4	1.39	99.27 ± 3.08	3.10

#### Selectivity

By contrasting blank human plasma samples from six distinct sources with blank human plasma samples treated with analytes at the LLOQ, selectivity was demonstrated. Since the peak regions of coeluting substances did not reach 20% of the analyte at the LLOQ, as shown in Fig. 2a and b, there was no evidence that endogenous matrix components had an impact on the peak resolution of the medications under investigation.

#### Carryover and matrix effects

After the ULOQ sample was injected, blank samples were injected to measure the carry-over effect. The results showed that after injecting the blank sample, there was no interference. Following injection of the LQC and HQC samples made from six various human plasma samples to evaluate matrix effects, the %RSD for paracetamol, rivaroxaban, and ceftriaxone at the LQC were 6.328, 4.163, and 12.033, respectively, and the %RSD for paracetamol, rivaroxaban, and ceftriaxone at the HQC were 1.947, 7.576, and 3.350, respectively, as shown in Table 3. Human plasma with the LQC and HQC samples did not exhibit any discernible matrix impact.

**Table 3:** Results of the matrix effects of the investigated analytes in human plasma.

Analytes	Level	Concentration added (µg/mL)	Matrix effect	
			Mean ± SD	RSD (%)
Paracetamol	LQC	3.0	89.01 ± 5.633	6.328
	HQC	12.5	88.33 ± 1.719	1.947
Rivaroxaban	LQC	0.3	101.37 ± 4.220	4.163
	HQC	7.5	103.64 ± 7.852	7.576
Ceftriaxone	LQC	3.0	101.33 ± 12.194	12.033
	HQC	12.5	100.89 ± 3.380	3.350

#### Stability

The freeze‒thaw and bench-top stabilities of the analytes in plasma were evaluated, and the results are displayed in Table 4.

**Table 4:** Results of freeze–thaw stability and bench top stability of the investigated analytes in human plasma.

Analytes	Level	Concentration added (µg/mL)	Freeze–thaw stability		Bench top stability	
			Mean ± SD	RSD (%)	Mean ± SD	RSD (%)
Paracetamol	LQC	3.0	94.21 ± 0.935	0.992	99.34 ± 0.826	0.832
	HQC	12.5	91.44 ± 3.071	3.359	96.59 ± 1.547	1.602
Rivaroxaban	LQC	0.3	108.79 ± 6.169	5.671	101.84 ± 0.427	0.419
	HQC	7.5	108.54 ± 4.028	3.711	104.38 ± 0.344	0.330
Ceftriaxone	LQC	3.0	99.81 ± 10.889	10.910	94.33 ± 7.819	8.289
	HQC	12.5	101.22 ± 3.445	3.404	97.14 ± 0.431	0.444

#### System suitability

To ascertain whether the instrumental system was operating effectively, system suitability testing was carried out. The parameters are clearly within the acceptable ranges, as shown in Table 5, demonstrating the success of the devised HPLC technique in effectively separating the examined analytes and demonstrating its high validity and reliability. Table 5 provides the results of the system suitability test^27^.

**Table 5:** System suitability parameters for the analysis of Paracetamol, Rivaroxaban, and ceftriaxone using the proposed HPLC method.

Parameters	Value			Reference^31^
	Paracetamol	Rivaroxaban	Ceftriaxone	
Capacity factor (K’)	4.38	5.31	20.54	K’ > 2
Tailing factor (T)	0.92	0.97	1.02	T ≤ 2
Number of theoretical plates (n)	2116	3285	17,423	*N* > 2000
Height equivalent to theoretical plates (HETP)	0.118	0.076	0.014	The smaller the value, the higher the column efficiency
selectivity (ɑ)	–	1.212	3.868	ɑ > 1
Resolution (RS)	–	3.88	3.0	RS > 1.5

### Method application in spiked human plasma

Utilizing the new, verified approach, the target analytes in plasma were measured. Table 6 presents the results of the application as percentage recoveries. Because the recommended method produces average recoveries between 85% and 115%, it can be used to quantitatively test target analytes with satisfactory results.

**Table 6:** Determination of the investigated analytes in human plasma using the proposed HPLC method.

Analyte	Spiked conc. (µg/ml)	Found conc. (µg/ml)	Recovery%
Paracetamol	4.0	4.16	103.98
	8.0	7.23	90.39
	13.0	12.29	94.55
Mean ± SD	96.31 ± 6.960		
Rivaroxaban	0.2	0.21	105.81
	4.0	3.89	97.27
	7.0	6.38	91.11
	9.0	8.81	97.92
Mean ± SD	98.03 ± 6.029		
Ceftriaxone	4.0	4.14	103.61
	8.0	8.23	102.90
	13.0	13.04	100.29
Mean ± SD	102.27 ± 1.747		

### Greenness assessment of the proposed method

Four metrics, namely, the national environmental methods index (NEMI), analytical eco-Scale (ESA), green analytical procedure index (GAPI), and analytical greenness index (AGREE) were adopted to assess the method’s greenness. Combination of AGREE tool with GAPI tool is very useful in the greenness assessment of analytical methods in biological fluids and tissues^32^.

#### National environmental methods index (NEMI)

**Fig. 3:**
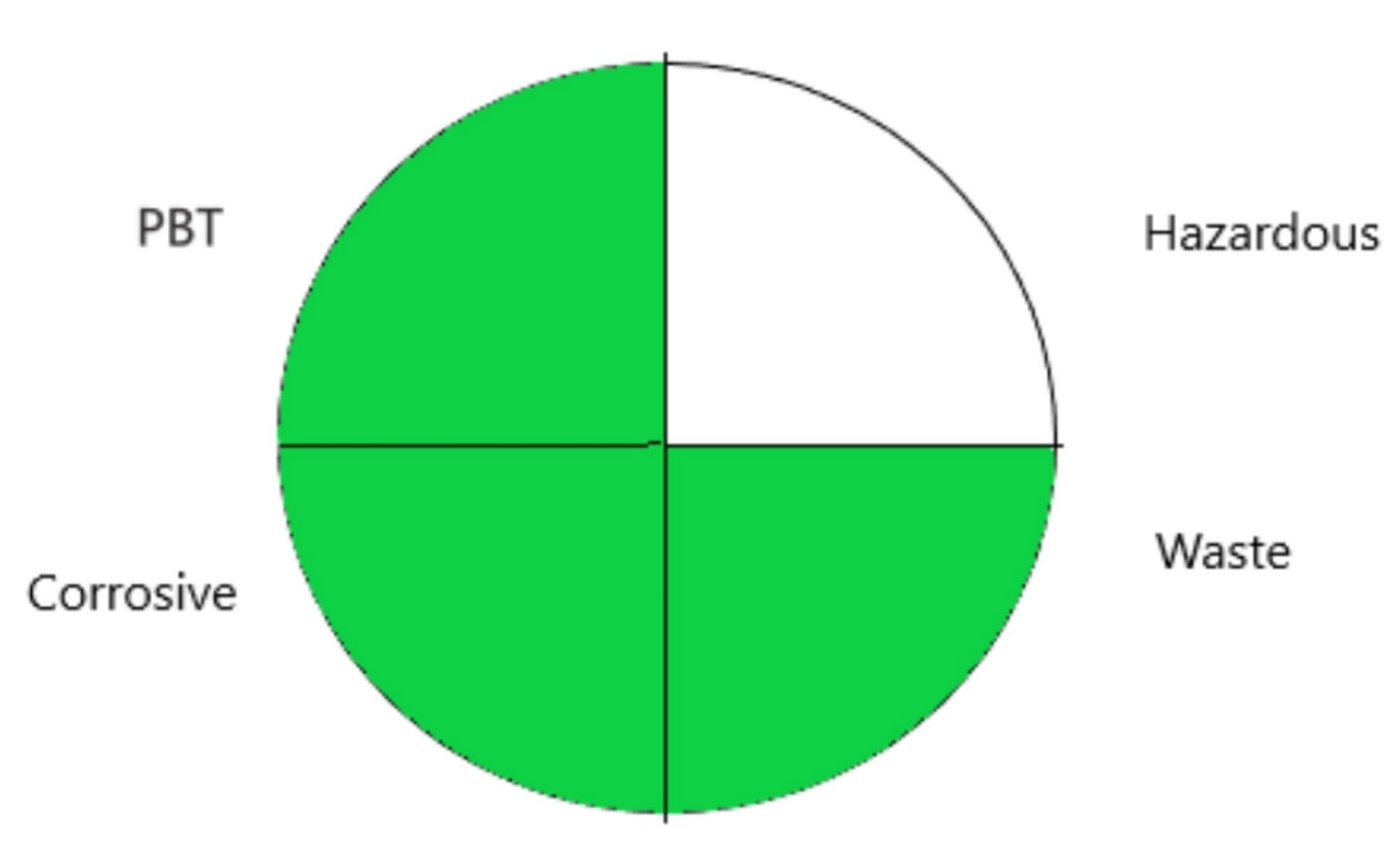
NEMI pictogram for assessment of “greenness” of the proposed method.

It is a metric system based on a circle that is divided into four sections; each sector stands for a certain criterion (hazardous, corrosive, persistent, bio accumulative and toxic chemicals (PBT), and waste prevention)^33^. The quarter is colored green when the value of a criterion is met; otherwise, it is left empty. The utilized reagents are not defined as PBT by the Environment Protection Agency’s Toxic Release Inventory (EPA-TRI). Since methanol, and acetonitrile are well-known toxic substances listed on the TRI list^34^, the hazardous quarter was left empty. Since the pH of the mobile phase was between 2 and 12, there was no corrosiveness that could have endangered the environment during analysis, and as a result, the pH quarter was green. Less than 50 g of waste was produced, making the waste quarter green as well. The results of NEMI are shown in Fig. 3.

#### The analytical eco-scale tool (ESA) for evaluating the proposed method’s greenness

According to eco-scale estimations, the optimal green procedure receives a score of 100^33^. After that, reagents, instruments, energy, and waste generation are penalized. These points, along with the analytical eco-scale scores, are displayed in Table 7. Finally, the eco-scale score was determined by deducting the penalty point from the ideal 100 points. This analytical method yielded a score of 81, which is above the 75th percentile in this metric system, indicating that this method is highly environmentally friendly.

**Table 7:** Penalty points for the analytical eco-scale assessment (ESA) of the proposed HPLC method.

Hazard	Penalty points
Reagents	
Acetonitrile	4
Methanol	6
Water	0
Instruments	
Energy and occupational hazard	1
Waste	8
Total penalty points	19
Analytical eco-scale total score	81

#### Green analytical procedure index (GAPI)

**Fig. 4:**
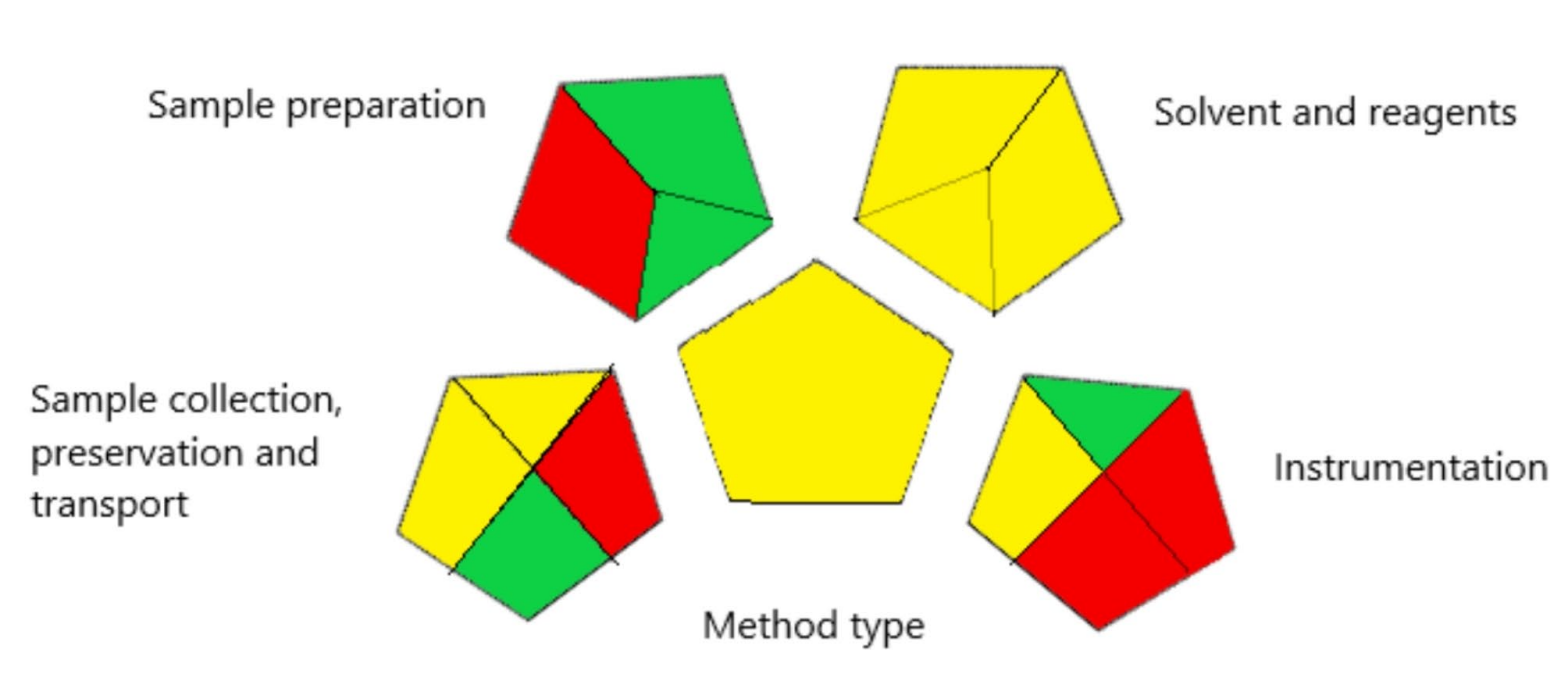
Green analytical procedure index (GAPI) of the proposed method.

The green analytical process index (GAPI), developed by Plotka-Wasylka^35^ is a way of measuring greenness that is relatively new. It monitors every step of the procedure, from sample collection to waste processing^36^. It gives 15 elements to be assessed in three color-coded levels: green for low environmental threat, yellow for intermediate environmental danger, and red for bad impact. The suggested method fulfilled the majority of GAPI criteria with just four red zones as shown in Fig. 4. The first was brought on by the collecting of samples offline, the second by precipitating plasma proteins in a non-green solvent, the third and fourth by the lack of options for waste treatment that is more than 10 mL. Regarding the yellow regions, regions 3 and 4 are yellow because the human plasma must be transported from the location of collection to the laboratory and stored under normal conditions. Region 5 is colored yellow since the method requires simple preparation such as filtration, while regions 9, 10, and 11 are yellow because the amount of solvents used is more than 10 mL with moderate health and environmental effects, according to the National Fire Protection Association (NFPA). Region 12 is yellow because HPLC uses less than 1.5 KWh per sample.

#### Analytical GREEnness metric method and Software (AGREE) for evaluating the proposed method’s greenness

**Fig. 5:**
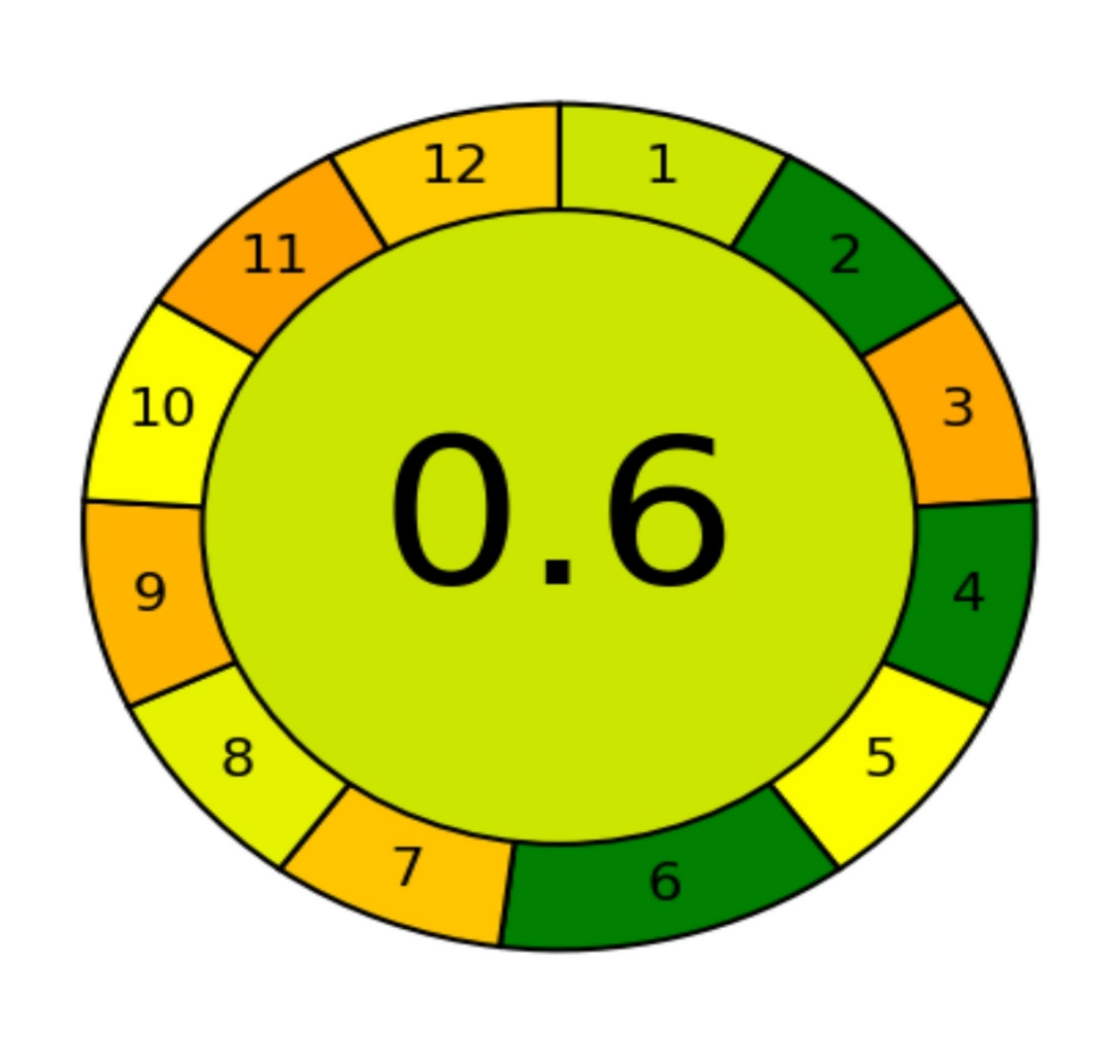
Result of the AGREE green profile assessment software of the proposed method.

An easy-to-understand and instructional score is produced by the comprehensive, flexible, and straightforward Analytical GREEnness software^37^. Using the 12 principles of green analytical chemistry (SIGNIFICANCE) as a guide, the evaluation standards were transformed into a common 0–1 scale. The ultimate score is established based on the SIGNIFICANCE principles. Upon completion, the user-assigned weights, the total score, and the analytical technique’s performance on each criterion are displayed in a pictogram. With an overall score of 0.6, as illustrated in Fig. 5, our recommended approach is deemed to be green.

The main drawback of GAPI is complexity compared to NEMI and ESA. AGREE has the merits of simplicity and automation over GAPI^38^. Recommendations are made for ESA, GAPI, and AGREE tools, which provide reliable and precise results about the greenness of the method.

## Conclusion

For the effective simultaneous quantification of paracetamol, rivaroxaban, and ceftriaxone in human plasma employed in COVID-19 management, a suggested isocratic RP-HPLC combined with DAD approach was developed. The proposed method offers good sensitivity over wide concentration ranges as well as good accuracy and precision. For each of the three target analytes in human plasma, distinct calibration curves were created. The FDA requirements were followed for the bioanalytical validation of the procedure. The suggested approach was found to be eco-friendly, straightforward, quick, and sensitive; therefore, it is advised for the measurement of the investigated analytes in human plasma. Furthermore, it paved the road for developing other methods for simultaneous analysis of the studied combination in different matrices such as urine, and saliva. Implementation of greener solvents, while taking in consideration shortening run time and achieving higher sensitivity, could be addressed as well. Although HPLC is a powerful separation tool, its limitations may include being a costly technique due to the need of numerous expensive organic solvents, and regular maintenance.

## Electronic supplementary material

Below is the link to the electronic supplementary material.

Supplementary Material 1

## Data Availability

All data generated or analysed during this study are included in this published article [and its supplementary information files].
