# Supplementary material for: A bioanalytically validated RP-HPLC method for simultaneous quantification of rivaroxaban, paracetamol, and ceftriaxone in human plasma: a combination used for COVID-19 management
Source: Sci Rep. 2024 Oct 28;14:25693. doi: 10.1038/s41598-024-75729-y (PMC11514257; doi:10.1038/s41598-024-75729-y)
Supplement: Supplementary file 1 — Supplementary Material 1 [file 41598_2024_75729_MOESM1_ESM.docx]

**Highlights**

- The proposed method is the first chromatographic method for simultaneous determination of rivaroxaban with paracetamol and ceftriaxone as co-administered drugs in Covid-19 management in human plasma samples.
- The developed method has been bioanalytically validated in accordance with FDA guidelines and showed excellent accuracy and precision for simultaneous quantification of the studied drugs in human plasma samples.
- The suggested method was found to be green with a value of 81 and 0.6 for the analytical eco-scale and analytical greenness assessment (AGREE), respectively.
